# Oral vitamin B12 for patients suspected of subtle cobalamin deficiency: a multicentre pragmatic randomised controlled trial

**DOI:** 10.1186/1471-2296-12-2

**Published:** 2011-01-13

**Authors:** Bernard Favrat, Paul Vaucher, Lilli Herzig, Bernard Burnand, Giuseppa Ali, Olivier Boulat, Thomas Bischoff, François Verdon

**Affiliations:** 1 Department of Ambulatory Care and Community Medicine, University of Lausanne, Bugnon 44, CH-1011 Lausanne, Switzerland; 2 Institute of General Medicine, University of Lausanne, Bugnon 44, CH-1011 Lausanne, Switzerland; 3 Institute of Social and Preventive Medicine, University of Lausanne, Bugnon 17, CH-1005 Lausanne, Switzerland; 4 Laboratory of Clinical Chemistry, Hospices-CHUV, Bugnon 46, CH-1011 Lausanne, Switzerland

## Abstract

**Background:**

Evidence regarding the effectiveness of oral vitamin B12 in patients with serum vitamin B12 levels between 125-200 pM/l is lacking. We compared the effectiveness of one-month oral vitamin B12 supplementation in patients with a subtle vitamin B12 deficiency to that of a placebo.

**Methods:**

This multicentre (13 general practices, two nursing homes, and one primary care center in western Switzerland), parallel, randomised, controlled, closed-label, observer-blind trial included 50 patients with serum vitamin B12 levels between 125-200 pM/l who were randomized to receive either oral vitamin B12 (1000 μg daily, N = 26) or placebo (N = 24) for four weeks. The institution's pharmacist used simple randomisation to generate a table and allocate treatments. The primary outcome was the change in serum methylmalonic acid (MMA) levels after one month of treatment. Secondary outcomes were changes in total homocysteine and serum vitamin B12 levels. Blood samples were centralised for analysis and adherence to treatment was verified by an electronic device (MEMS; Aardex Europe, Switzerland). Trial registration: ISRCTN 22063938.

**Results:**

Baseline characteristics and adherence to treatment were similar in both groups. After one month, one patient in the placebo group was lost to follow-up. Data were evaluated by intention-to-treat analysis. One month of vitamin B12 treatment (N = 26) lowered serum MMA levels by 0.13 μmol/l (95%CI 0.06-0.19) more than the change observed in the placebo group (N = 23). The number of patients needed to treat to detect a metabolic response in MMA after one month was 2.6 (95% CI 1.7-6.4). A significant change was observed for the B12 serum level, but not for the homocysteine level, hematocrit, or mean corpuscular volume. After three months without active treatment (at four months), significant differences in MMA levels were no longer detected.

**Conclusions:**

Oral vitamin B12 treatment normalised the metabolic markers of vitamin B12 deficiency. However, a one-month daily treatment with1000 μg oral vitamin B12 was not sufficient to normalise the deficiency markers for four months, and treatment had no effect on haematological signs of B12 deficiency.

## Background

Vitamin B12 deficiency (less than 150 pM/l) is common in elderly people, with the reported prevalence ranging from 15% to 20% [1,2]. Most patients show no evidence of megaloblastic anaemia; however, these patients are still at risk for neurological abnormalities [3]. Furthermore, vitamin B12 deficiency leads to hyperhomocysteinemia, an independent risk factor for ischemic heart disease [4] and dementia [5]. Comparisons between patients with known complications and "normal" control patients [6] have produced several definitions of cobalamin deficiency. Over the past 15 years, the definition of vitamin B12 deficiency has included determination of two metabolites due to their favourable sensitivities and specificities: serum methylmalonic acid (MMA) and homocysteine (Hcys) [7,8]. Over the past few decades, a number of publications and two randomised controlled trials have demonstrated that oral B12 is as efficacious as intramuscular injection, especially for normalising metabolic markers of cobalamin deficiency [9-13], and is also cost-effective [14].

Nevertheless, there is a lack of randomised placebo-controlled trials to validate the use of oral vitamin B12 therapy [15]. Furthermore, few randomised placebo-controlled trials have evaluated the biological impact of oral therapy (1000 μg/d) in general practice for borderline serum vitamin B12 concentrations (125-200 pM/l) among patients without pernicious anaemia [16,17]. Our objective was to evaluate the efficacy of oral cobalamin in reducing MMA levels in patients suspected of vitamin B12 deficiency but with borderline vitamin B12 concentrations. This type of study is important for helping to determine whether the MMA level "indicates or predicts a clinical condition in need of treatment" [18].

## Methods

A pragmatic [19,20], placebo-controlled, randomised controlled trial with a four-month follow-up period was conducted by 16 general practitioners in the western part of Switzerland between October 2002 and September 2004. The study protocol was approved by the ethics review committee for clinical research of the Department of Internal Medicine, University of Lausanne, and was registered in the Current Controlled Trial Database (ISRCTN 22063938).

### Participants

Physicians enrolled patients from private practices (13 general practitioners), an academic primary care centre (counted as one general practitioner), and nursing homes (two general practitioners). Patients in whom physicians suspected B12 deficiency based on clinical parameters were asked to participate. Patients who were suspected of having cobalamin deficiency met at least one of the following inclusion criteria: history of cobalamin deficiency, red cell macrocytosis (>99 fl), or neurological or psychiatric symptoms (or both) defined as having three or more positive responses to the symptoms described in Table 1.

**Table 1:** Reasons for patient eligibility reported by physicians (N = 49*)

Symptoms	Oral B12 (N = 26) N(%)	Placebo (N = 23*) N (%)
"Do you have a pins and needles feeling in your feet?" or "Do you have a compelling urge to move your legs in the evening or at night?"	10 (38%)	7 (30%)
"Have you recently felt unsteady when walking for reasons other than your rheumatism or after having an accident?"	5 (19%)	3 (13%)
"During the past month have you often been bothered by feeling down, depressed, or hopeless?" and "During the last month, have you often been bothered by having little interest or pleasure in doing things? [41]	7 (27%)	8 (35%)
"Did you feel that you were losing your memory or has someone mentioned this to you lately?"	5 (19%)	4 (17%)
"Has your character changed lately or has someone mentioned this to you?"	2 (8%)	3 (13%)
"Has it been more difficult for you to perform your usual activities, such as reading a book, watching TV, writing, paying bills, doing your housework, etc.?"	5 (19%)	6 (26%)
Anemia detected (blood formula)	4 (15%)	6 (26%)
Macrocystosis (blood formula)	4 (15%)	5 (21%)

* One questionnaire was not returned by the physician.

Consenting patients with serum vitamin B12 levels equal to or greater than 125 pM/l but equal to or less than 200 pM/l were included. Exclusion criteria included folate deficiency, renal insufficiency, and folate or vitamin B12 treatment during the preceding six months. For ethical reasons, patients with vitamin B12 levels less than 125 pM/l after one month received oral vitamin B12 supplementation for one month. Written informed consent was obtained from all patients before screening for vitamin B12 deficiency. Details of refusal, exclusion, dropouts, and missing data were collected when available. Blood samples and baseline values were collected before treatment allocation. All blood samples were centralised and analysed using a single analysis method.

### Treatment, randomisation, blinding, and adherence to therapy

Participants received either 1000 μg oral vitamin B12 (cobalamin) or placebo daily for four weeks. An independent pharmacist delivered active or placebo pills according to a prior, simple computer-generated randomisation list. The active and placebo pills were similar in appearance and taste and were given in a similar container. Patients, caregivers, investigators, and the statistician were blinded to treatment until the end of the trial. Each drug package was coded with a unique number according to the randomisation schedule, then sent to the relevant practice. The codes were held by the pharmacists and remained unbroken until the analysis was completed. Patients were asked not to take any other vitamin supplements, and the treating physician verified this at the one- and four-month follow-up visits.

### Outcomes

The principle outcome was the relative difference in serum MMA levels at baseline and after one month of treatment. Responders were defined as those participants exhibiting more intra-individual variation in MMA (a decrease in the serum MMA level greater than 0.076 μmol/l) than that caused by random variation [21]. Secondary outcomes were changes in serum vitamin B12 levels, total Hcys levels, and clinical improvement based on symptoms and physical signs at the one- and four-month follow-up visits. Changes in MMA levels measured at baseline and after four months were also determined. The relative improvement toward the population mean value and the number of patients needed to treat (NNT) were reported as secondary outcomes for MMA. Haematological improvement was measured for mean corpuscular volume and hematocrit. Cognitive changes were assed using the Mini Mental State Examination (scale ranging from 30 to 0).

### Measurements and laboratory methods

Medical visits and blood sampling were performed by physicians at baseline and after one and four months. Participants participated in a structured interview to record socio-demographic variables, medical history, complaints, the reason why the physician suspected vitamin B12 deficiency, medication, importance of neurological or psychiatric signs, and state of health.

Blood samples were drawn by standard antecubital venipuncture from patients who were not fasting. Two separate tubes were collected; all tubes were centralised for analysis in a single laboratory. Samples were centrifuged and serum concentrations of MMA, Hcys, creatinine, vitamin B12, and folate were measured. Renal insufficiency was defined as a serum creatinine level greater than 97 μmol/l, folate insufficiency as a serum value below 7 nmol/l, and red cell macrocytosis as a value greater than 99 fl. Haematological parameters were measured by an automated analyser (Sysmex XE-2100; Sysmex corporation, Hyogo, Japan). Serum creatinine levels were measured by the Jaffé kinetic method at 37°C (Modular ANALYTICS system; Roche Diagnostics, Basel, Switzerland). Serum vitamin B12 and folate levels were measured by a quantitative radioimmunoassay using purified intrinsic factor and purified folate-binding protein. MMA levels in serum were determined by gas chromatography and mass spectrometry with isotopic dilution. Analytical performance was assessed by internal and external quality controls (ERNDIM; http://www.erndimqa.nl/). Typical coefficients of variation were less than 3.8% at 0.39 μmol/l (N = 55) and less than 3.7% at 1.75 μmol/l (N = 61). Total Hcys levels in serum were quantified by high performance liquid chromatography with fluorimetric detection [22]. Typical coefficients of variation were less than 5.2% at 5.5 μmol/l (N = 120) and less than 3.3% at 16.4 μmol/l (N = 143). Adherence to treatment was verified by an electronic device (MEMS; Aardex Europe, Switzerland) that recorded the date and time of every opening of the pill container. Unused pills were also counted. Adherence was quantified by calculating the percentage of days that the pill container was opened once.

### Statistical methods

Our study was powered to detect a mean decrease in MMA values of 0.2 μmol/l in the treatment group as compared with 0.05 μmol/l in the placebo group. Expecting a standard deviation of 0.15 μmol/l with the significance level set at p < 0.05 and power set at 0.8, the estimated sample size was 16 participants in each group. Based on previous data [23], we estimated that the proportion of responders (as determined by a change in MMA) would be 10% in the placebo group and at least 50% in the intervention group, necessitating the inclusion of 25 patients in each group.

We performed the principal analysis according to intention to treat (all patients remained in their initially assigned arm). The planned measure of magnitude of effects was absolute change difference from baseline between the treatment and placebo arms. This difference was determined by computing the least square means of differences with linear regression, adjusting for baseline value. Robust standard error [24] was used to take heteroscedasticity into consideration. To control for the observed lack of homogeneity of variance for different baseline values, we also computed the improvement from baseline in proportion to what would have been expected had the values become normal (relative improvement). This secondary measure of intervention effect was the relative improvement difference between arms (RΔ%). Based on previously published information, we considered the healthy population's mean MMA value to be 0.17 μmol/l [25], the mean Hcys value to be 10.2 μmol/l [25], and the mean vitamin B12 value to be 375 pM/l [26]. If the values changed to exceed the mean population value, the measure of effect was limited to 1.0. If no improvement was detected at follow-up, or if the baseline values were already normal, the measure of effect was considered null. Student's *t*-test was used to evaluate the significance of the observed difference between those patients receiving B12 and those receiving placebo. No measures were taken to control the overall type I error rate because outcomes were expected to be highly correlated to each another. The NNT was calculated considering dropouts and missing data as non-responders. We set the alpha level to 0.05 and calculated 95% confidence intervals (CIs). All statistical analyses were performed using Stata 10.0 (StataCorp LP, College Station, TX, USA).

## Results

**Figure 1:**
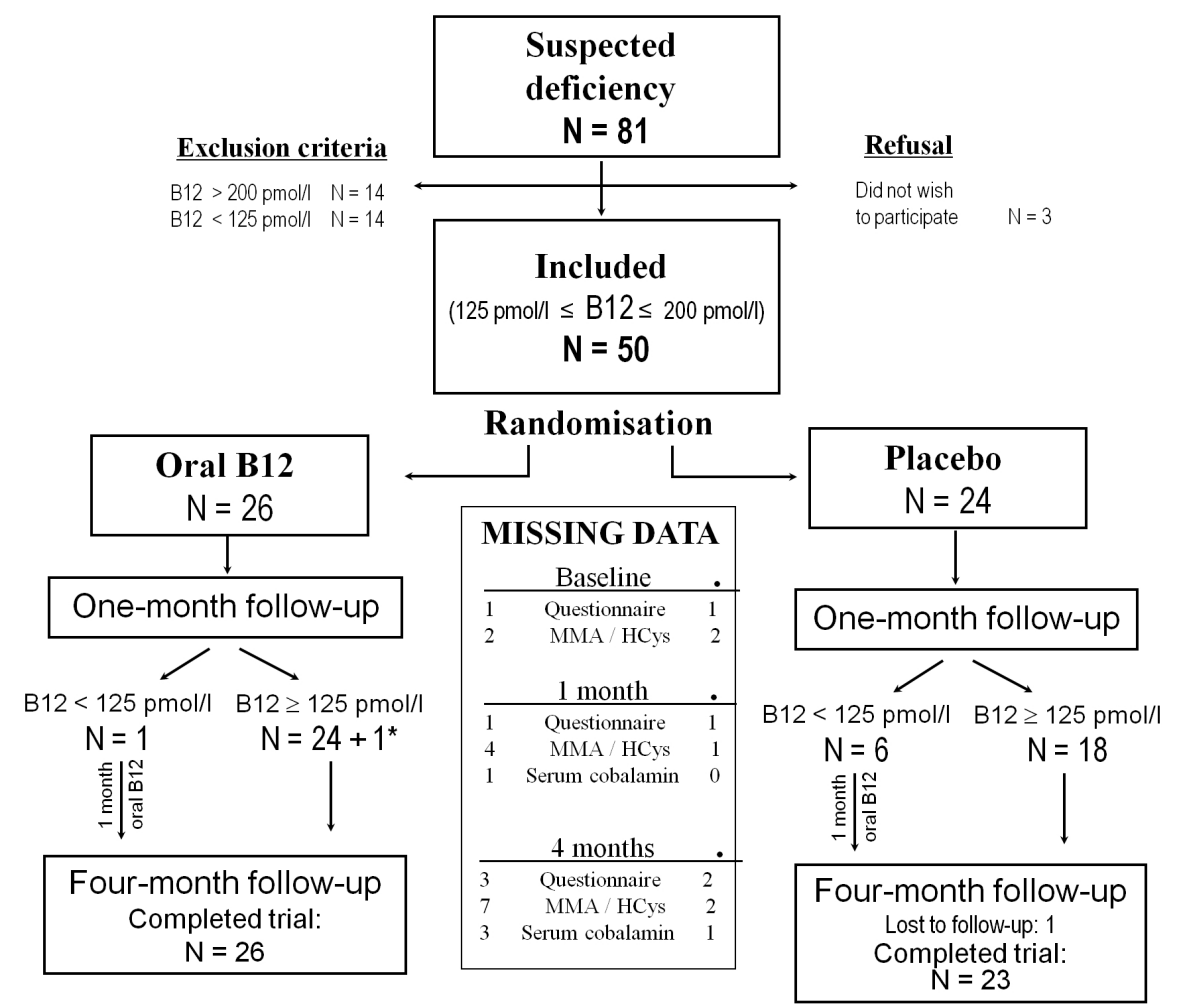
**Flow chart of the study design**. * B12 concentration was not available for one patient who was assumed not to be deficient. Hcys: homocysteine, MMA: methylmalonic acid.

Vitamin B12 deficiency was suspected in 81 patients. Three patients refused to participate (4%) without providing reasons, 14 (17%) had B12 serum values less than 125 pM/l and were given B12 treatment, and 14 (17%) had serum values greater that 200 pM/l and were excluded. The 50 remaining patients were randomly assigned (Figure 1) to the placebo arm (N = 24) or the experimental arm (N = 26). The symptoms that most frequently made the physician suspect B12 deficiency were paraesthesia and depression (Table 1). Age, sex, and other characteristics were similar between the groups (Table 2). Doses and proportion of days with correct medication were 27.4 intakes and 93.5% of days for the oral B12 group and 28.4 intakes and 94.4% of days for the placebo group. Seven patients (one from the treatment group) had vitamin B12 levels less than 125 pM/l at one month and received oral vitamin B12 supplementation for one extra month. These patients were included in the intention to treat analysis. One questionnaire was not completed by the physician. Transport inconvenience prevented 18/145 blood samples (from 13 patients) from being analysed for metabolites. Six of 50 electronic devices used to measure treatment compliance were not returned; thus, adherence data were missing for these patients. We observed no relevant side effects of treatment during the four-month follow-up period. Two adverse events were reported, both in the intervention group. One patient was hospitalised for psychiatric reasons and one was hospitalised to receive a blood transfusion. Physicians considered both of these events unrelated to vitamin B12 administration.

**Table 2:** Baseline characteristics of study participants

	Oral B12 (N = 26)	Placebo (N = 24)
**Gender (Female)**	14 (53.8%)	13 (54.2%)
n (%)		
**Age**		
Mean (SD)	69.6 yrs (SD = 18.8)	68.6 yrs (SD = 18.5)
Median (range)	76 yrs (31 - 91)	75 yrs (18 - 88)
**Serum B12**		
Mean (SD)	164 pM/l (SD = 24)	154 pM/l (SD = 20)
Median (range)	164 pM/l (127 - 203)	150 pM/l (126 - 191)
**Serum MMA**		
Mean (SD)	0.43 μmol/l (SD = 0.25)*	0.41 μmol/l (SD = 0.24)*
Median (range)	0.32 μmol/l (0.19 - 1.1)	0.31 μmol/l (0.15 - 0.92)
**MMA ≥ 0.26 μmol/l**		
n (%)	18 (83.3%)*	16 (72.7%)*
**Serum HCys**		
Mean (SD)	18.3 μmol/l (SD = 6.6)*	15.0 μmol/l (SD = 5.3)*
Median (range)	18.1 μmol/l (9.8 - 31.5)	14.3 μmol/l (7.5 - 27.1)
**Hematocrit**		
Mean (SD)	40.3% (SD = 4.2)^†^	39.5% (SD = 4.6)
Median (range)	39.5% (32 - 46)	40% (27 - 47)
**Mean corpuscular volume**		
Mean (SD)	91.2 fl (SD = 9.2)^†^	92.6 fl (SD = 5.1)
Median (range)	90 fl (63 - 100)	92 fl (81 - 111)
**Serum creatinine**		
Mean (SD)	96.4 μmol/l (SD = 27.9)	89.0 μmol/l (SD = 27.2)
Median (range)	86 μmol/l (60 - 160)	89 μmol/l (37 - 137)
**Serum folic acid**		
Mean (SD)	16.6 nmol/l (SD = 9.1)	19.2 nmol/l (SD = 10.9)
Median (range)	16.2 nmol/l (5.6 - 45.3)	14 nmol/l (5.4 - 33)

Hcys = homocysteine, MMA = methylmalonic acid, SD = standard deviation

* Transport inconvenient generated missing total at random data; samples were not analysed for 2 patients in the oral B12 group and for 2 patients in the placebo group.

^† ^One physician did not report results from the blood formula onto the case report form. Data were unvailable for one patient in the oral B12 group.

**Figure 2:**
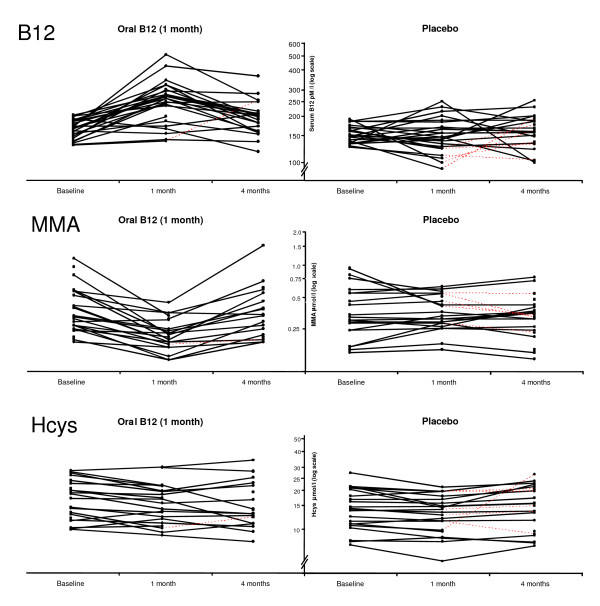
**Changes in metabolic values**. Red dotted lines correspond to patients from the placebo group who received vitamin B12 for one month at the one-month follow-up. Hcys: homocysteine, MMA: methylmalonic acid.

At baseline, 21.7% of patients had MMA levels that were considered normal (less than 0.26 μmol/l). Serum concentrations of metabolites at baseline, one month, and four months appear in Figure 2. A significant treatment effect was observed on surrogate values of serum vitamin B12 after both one and four months, and on MMA values at one month (Table 3). Per-protocol analysis also confirmed the absence of a difference in mean MMA concentrations between the placebo and treatment groups at four months (-0.02 μmol/l; 95% CI -0.16 to 0.13; p = 0.832). We also measured the relative improvement effect toward the healthy population's mean value (Table 4). At one month, patients undergoing vitamin B12 treatment decreased their mean deficit by 48.7% (95% CI 29.0 to 68.3) over placebo. Finally, the NNT for improving MMA serum concentration at one month was 2.6 patients (95% CI 1.7 to 9.4).

**Table 3:** Different outcomes in the oral B12 treatment group and the placebo group after one and four months

	Oral B12		Placebo		Impovement from baseline between oral B12 and placebo*	
Biological marker	1 month Mean (SD); N	4 months Mean (SD); N	1 month Mean (SD); N	4 months Mean (SD); N	1 month* Δ (CI95%; p-value)	4 months* Δ (CI95%; p-value)
MMA (μmol/l)	0.23 (0.08); 22	0.41 (0.30); 19	0.37 (0.14); 23	0.35 (0.16); 22	- 0.13 (CI95% -0.19 to -0.06; p < 0.001)	0.03 (CI95% -0.12 to 0.17; p = 0.686)
Serum cobalamin (pM/l)	263.4 (89.8); 25	202.6 (56.3); 23	154.5 (41.1); 24	162.9 (39.8); 23	101.6 (CI95% 60.1 to 143.2; p < 0.001)	35.0 (CI95% 6.4 to 63.5; p = 0.018)
Hcys (μmol/l)	16.5 (6.1); 22	17.1 (7.5); 19	13.9 (4.3); 23	15.6 (5.8); 22	0.04 (CI95% -1.2 to 1.3; p = 0.950)	-1.0 (CI95% -4.0 to 2.0; p = 0.502)
Hematocrite (% red cells)	39.6 (4.1); 26	40.1 (4.0); 26	39.7 (4.6); 24	39.4 (4.6); 22	-0.4 (CI95% -1.7 to 0.8; p = 0.502)	0.5 (CI95% -1.0 to 2.1; p = 0.475)
MCV (fl)	89.8 (6.9); 26	89.0 (7.0); 26	92.8 (7.0); 24	92.6 (7.6); 22	-0.4 (CI95% -2.2 to 1.4; p = 0.674)	-0.1 (CI95% -2.3 to 2.2; p = 0.950)
MMSE (score 0-30)	-	27.8 (2.3); 26	-	28.1 (2.2); 21	-	-0.4 (CI95% -1.3 to 0.6; p = 0.432)

* Least square means were computed using linear regression with robust standard error to take heteroscedasticity into account. Each value at 1 or 4 months was introduced separately as the dependent variable, with group allocation and baseline value as independent variables. Interpretation of these magnitudes is nevertheless restricted. Variance is not constant across baseline values (heteroscedasticity). Effects are more important for patients with initial higher levels of deficiency. Hcys = homocysteine, MMA = methylmalonic acid, MMSE= Mini Mental State Examination.

**Table 4:** Differences in relative improvement* effects toward the healthy population's mean value between the oral B12 treatment and placebo group (RΔ%) at 1 and 4 months

	Relative improvement effect towards healthy population's mean value	
Biological marker	1 month RΔ% (CI95%; p-value)	4 months RΔ% (CI95%; p-value)
MMA (μmol/l) *Reduction*	48.7% (CI95% 29.0:68.3; p < 0.001)	0.2% (CI95% -17.8:18.3; p = 0.979)
Serum cobalamin (pM/l) *Increase*	26.9% (CI95% 13.9:39.8; p < 0.001)	14.1% (CI95% -3.1:31.3; p = 0.105)
Hcys (μmol/l) *Reduction*	1.8% (CI95% -16.4:20.0; p = 0.844)	6.7% (CI95% -11.3:24.7; p = 0.455)

* Improvement from baseline compared to what would have been expected had the values become normal (MMA = 0.17 μmol/l, vitamin B12 = 375 pM/l, and Hcys = 10.2 μmol/l).

## Discussion

This study has demonstrated that in primary care patients with nonspecific symptoms and borderline serum vitamin B12 levels, serum MMA levels were corrected more often in patients receiving one month of oral cobalamin therapy than in patients receiving one month of placebo. However, the benefit to the MMA level disappeared after three additional months without cobalamin therapy.

Although treatment has been deemed necessary regardless of laboratory results for patients with signs of severe cobalamin deficiency [27,28], in general practice one might question the impact of treatment for patients with nonspecific symptoms suspected to be related to cobalamin deficiency. The definition of cobalamin deficiency remains imprecise; a definition based only on clinical symptoms lacks specificity up to advanced stages, except in cases of pernicious anaemia. Therefore, a serum vitamin B12 level below 125 μmol/l cannot be the sole criterion for defining cobalamin deficiency. No clear cut-off values have been defined, and publications have variously asserted that there are "grey areas" below 295 pM/l [29], 200 pM/l [30], or 250 pM/l [27].

The metabolic markers MMA and Hcys show promise as markers for improving the diagnosis of cobalamin deficiency [7,8,31]. Our randomised controlled trial showed an improvement in MMA levels and a nonsignificant fall in Hcys following oral B12 supplementation for one month (Table 3). For MMA, our results corroborate observations from three other pertinent trials [17,32] and one equivalent trial comparing oral to parenteral administration [12]. However, our study is the first to follow patients after cessation of treatment. Our findings suggest that one month of treatment is not enough to maintain MMA serum concentrations above borderline deficit. Furthermore, one month of treatment may not be sufficient to affect Hcys levels, as other studies have demonstrated improvement after treatment periods of three months [33]. The specificity of Hcys is considered low, which may also explain the low response of Hcys levels to vitamin B12 therapy [27]. Hcys levels are also influenced by lifestyle habits (coffee, alcohol, and smoking), renal function, genetic abnormalities, and folate deficiency.

In the primary care setting, the importance of subtle cobalamin deficiency and its related clinical impact remain under debate. Increased concentrations of MMA metabolites in patients without anaemia define subtle cobalamin deficiency [29]. The prognostic and clinical significances of this state are not clear [34]. Neuropathy, anaemia, and cognitive impairment are possible [15,35,36], and some observational studies have demonstrated a clinical benefit from treatment, including oral therapy [15,35]. However, Solomon found patients with clinical signs of cobalamin deficiency but normal levels of metabolic markers [34]. In a randomised controlled trial in community-dwelling subjects, Lewerin found that four months of oral 0.5 mg vitamin B12 in combination with folic acid and vitamin B6 normalised serum MMA and Hcys levels, but failed to improve movement and cognitive performance [37]. Hvas also reported limited clinical improvement following administration of vitamin B12 to patients with elevated MMA (0.4-2 μmol/l) levels [18,34,38]. However, controversy continues to surround the question of whether vitamin B12 supplements affect cognition [39]. In our study, one month of oral vitamin B12 significantly increased serum cobalamin levels, but the effect did not persist after three more months without supplementation. These results are unexpected, given the liver storage capacity for vitamin B12 and the quantity of B12 administered during this study, and the effectiveness of intermittent treatment remains uncertain. Surrogates alone may not provide sufficient evidence to assume clinical benefits of vitamin B12 supplementation. Further studies are required to assess the effects on clinical outcomes.

One limitation of our study lies in defining the population for which our results are applicable. Further randomised trials are still necessary to evaluate the prophylactic effect of oral B12 for preventing neurological manifestations [40]. Mishandling of blood samples resulted in the loss of some data. These unexpected events and other missing data were not included in our initial sample size estimation, limiting the power of our study. Most missing data were missing completely at random, our results were therefore not biased, and only the power of the study was diminished. Finally, the inclusion of essentially non-anaemic patients who are less likely to respond to vitamin B12 treatment may affect our ability to generalise our results to an anaemic, cobalamin-deficient population.

## Conclusion

Although oral B12 therapy evoked an important metabolic response, this response did not persist for an additional three months following cessation of therapy, causing us to question whether extending oral vitamin B12 treatment beyond one month would have a significant effect on the clinical manifestations of cobalamin deficiency. Whether correcting abnormal metabolic markers in hopes of improving clinical symptoms in patients with suspected borderline cobalamin deficiency is a clinically auspicious strategy remains under debate.

## List of abbreviations

Hcys: homocysteine; MMA: methylmalonic acid; NNT: number needed to treat; SD: standard deviation; RΔ%: relative reduction difference towards the healthy population's mean value

## Competing interests

BF, PV, LH, BB, GA, OB, TB, and FV all declare the absence of any financial or non-financial competing interests.

## Authors' contributions

BF, FV, LH, BB, GA, and TB designed the study. PV, BF, and FV analysed and interpreted the data. BF and PV drafted the manuscript. FV, LH, BB, GA, and TB revised and corrected the draft. All authors read and approved the final manuscript.

## Pre-publication history

The pre-publication history for this paper can be accessed here:

http://www.biomedcentral.com/1471-2296/12/2/prepub
